# Influence of an embedded quantum dot on the Josephson effect in the topological superconducting junction with Majorana doublets

**DOI:** 10.1038/srep23033

**Published:** 2016-03-14

**Authors:** Wei-Jiang Gong, Zhen Gao, Wan-Fei Shan, Guang-Yu Yi

**Affiliations:** 1 College of Sciences, Northeastern University, Shenyang 110819, China

## Abstract

One Majorana doublet can be realized at each end of the time-reversal-invariant Majorana nanowires. We investigate the Josephson effect in the Majorana-doublet-presented junction modified by different inter-doublet coupling manners. It is found that when the Majorana doublets couple indirectly via a non-magnetic quantum dot, only the normal Josephson effect occurs, and the fermion parity in the system just affects the current direction and amplitude. However, one magnetic field applied on the dot can induce the fractional Josephson effect in the odd-parity case. Next if the direct and indirect couplings between the Majorana doublets coexist, no fractional Josephson effect takes place, regardless of the presence of magnetic field. Instead, there almost appears the *π*-period-like current in some special cases. All the results are clarified by analyzing the influence of the fermion occupation in the quantum dot on the parity conservation in the whole system. We ascertain that this work will be helpful for describing the dot-assisted Josephson effect between the Majorana doublets.

Topological superconductor (TS) has received considerable experimental and theoretical attentions because Majorana zero-energy modes appear at the ends of the one-dimensional TS which can potentially be used for decoherence-free quantum computation^1^,^2^,^3^. In comparison with the conventional superconductor, the TS system shows new and interesting properties^4^,^5^. For instance, in the proximity-coupled semiconductor-TS devices, the Majorana zero modes induce the zero-bias anomaly^6^,^7^. A more compelling TS signature is the unusual Josephson current-phase relation. Namely, when the normal s-wave superconductor nano-wire is replaced by a TS wire with the Majorana zero modes, the current-phase relation will be modified to be 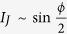 and the period of the Josephson current vs *ϕ* will be 4*π* (*ϕ* is the superconducting phase difference). This is the so-called the fractional Josephson effect^8^,^9^,^10^,^11^,^12^. Such a result can be understood in terms of fermion parity (FP). If the FP is preserved, there will be a protected crossing of the Majorana bound states at *ϕ* = *π* with perfect population inversion. As a result, the system cannot remain in the ground state as *ϕ* evolves from 0 to 2*π* adiabatically^13^,^14^.

Recently, the time-reversal invariant TSs, i.e., the DIII symmetry-class TSs^15^,^16^,^17^,^18^,^19^, have attracted extensive attentions^20^,^21^,^22^. In such TSs, the zero modes appear in pairs due to Kramers’s theorem, which is different from the chiral TSs. Consequently, for the time-reversal-invariant TS nanowire, two Majorana bound states will be localized at each end of it and form one Kramers doublet^23^,^24^. Since the Kramers doublet is protected by the time-reversal symmetry, it will drive some new and interesting transport properties, compared with the single Majorana zero mode. Up to now, many schemes have been proposed to realize the time-reversal-invariant Majorana nanowires, by using the proximity effects of *d*-wave, *p*-wave, *s* ± -wave, or conventional *s*-wave superconductors^25^,^26^,^27^,^28^,^29^,^30^. Meanwhile, physicists have begun to pay attention to quantum transport phenomena contributed by the Kramers doublet, and some important results have been reported^31^,^32^. For instance, in the Josephson junction formed by the Majorana doublet, the Josephson currents show different periods when the FP in this system is changed^32^. This exactly means the nontrivial role of the Majorana doublet in manipulating the quantum transport. However, for completely describing the transport properties contributed by the Majorana doublet, any new proposals are desirable.

In this work, we aim to investigate the influence of an embedded quantum dot (QD) on the current properties in the Josephson junction contributed by the Majorana doublets. Our motivation is based on the following two aspects. Firstly, QD is able to accommodate an electron and the average electron occupation in one QD can be changed via shifting the QD level. Thus, when one QD is introduced in the TS junction, the FP can be re-regulated and the fractional Josephson current can be modified^33^. Moreover, some special QD geometries can induce the typical quantum interference mechanisms, e.g., the Fano interference^34^, which are certain to play an important role in adjusting the fractional Josephson effect. Secondly, one QD can mimic a quantum impurity in the practical system, which is able to provide some useful information for relevant experiments. Our calculations show that when the Majorana doublets couple indirectly via a non-magnetic QD, only the normal Josephson effect takes place, irrelevant to the FP change. As finite magnetic field is applied on the QD, the fractional Josephson effect comes into being in the odd-FP case. On the other hand, when the direct and indirect couplings between the Majorana doublets coexist, no fractional Josepshon effect occurs despite the application of magnetic field on the QD, but in the odd-FP case, the current oscillation manner undergoes discontinuous change following the shift of QD level. The results in this work will be helpful for describing the QD-assisted Josephson effect between the Majorana doublets.

## Model

**Figure 1:**
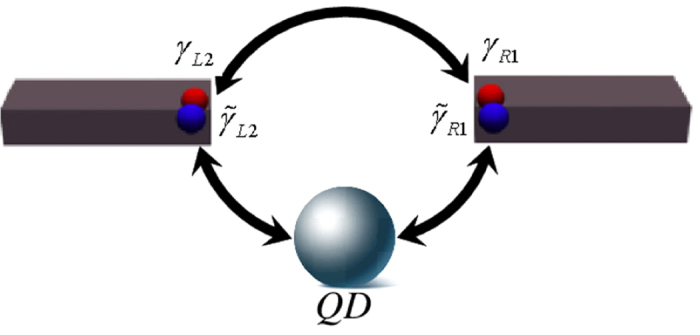
Schematic of Josephson junction formed by the direct coupling between the Majorana doublets and their indirect coupling via a QD.

The Josephson junction that we consider is formed by the direct coupling between the Majorana nanowires and their indirect coupling via a QD, as illustrated in Fig. 1. The particle tunneling process in this junction can be described by Hamiltonian *H*_*T*_ with


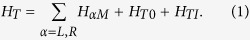


*H*_*αM*_ denotes the particle motion in the two Majorana nanowires. With the proximity-induced *p*-wave and *s*-wave superconducting pairs, the effective tight-binding Hamiltonian in the *α*-th nanowire can be written as^32^


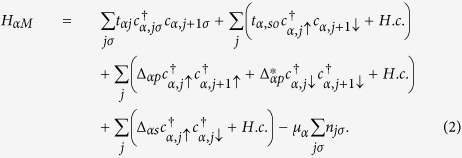



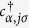
 and *c*_*α*,*jσ*_ (*σ* = ↑,↓ or ±1) are the electron creation and annihilation operators for the *j*-th site in the *α*-th nanowire. *t*_*jα*_ is the inter-site hopping energy and *t*_*α*,*so*_ represents the strength of spin-orbit coupling. Δ_*αp*_ and Δ_*αs*_ denote the energies of the *p*-wave and *s*-wave superconducting pairings, respectively. *μ*_*α*_ is the chemical potential in the *α*-th nanowire. Note that the hopping coefficients and the chemical potential are generically reonormalized by the proximity effect. The second term *H*_*T*0_ denotes the direct coupling between the two Majorana nanowires, which can be expressed as


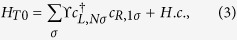


where *ϒ* is the direct coupling coefficient. Next, *H*_*TI*_ is to express the indirect coupling between the two Majorana nanowires due to the presence of an embedded QD (or a quantum impurity). Its expression can be given by


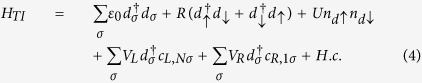


Here 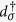 and *d*_*σ*_ are the electron creation and annihilation operators in the QD, and *ε*_0_ is the QD level. *R* denotes the strength of an effective magnetic field applied on the QD, and *U* denotes the intradot electron interaction with 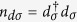. In addition, *V*_*α*_ is coupling coefficient between the QD and the *α*-th Majorana nanowire.

In order to discuss the Josephson effect in this junction, we have to deduce an effective Hamiltonian that reflects the direct and indirect couplings between the Majorana doublets. For this purpose, we define the Majorana operators


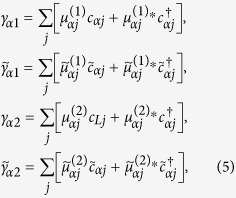


where *c*_*αj*_ = *s*_1_*c*_*α*,*j*↑_ + *s*_2_*c*_*α*,*j*↓_ is the new and spinless electron operator at the *j*-th site in the *α*-th site with 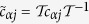. Using the above formulas, we can solve the electron operators in terms of Majorana and nonzero-energy quasiparticle operators. Reexpress the quasiparticles in terms of electron operators, we can interpret *c*_*LN*_, 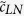, *c*_*R*1_, and 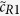 by


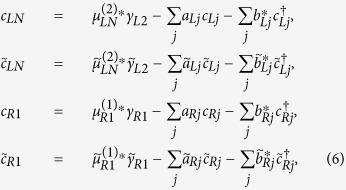


in which the normalization factor has been neglected. Besides, *a*_*αj*_, 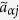 and *b*_*αj*_, 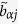 are expansion coefficients, originated from the quasiparticle operators other than the corresponding Majorana mode. Next, substitute Eq. (6) into the expression of *H*_*T*_, we can obtain the low-energy effective Hamiltonian of *H*_*T*_ in the case of infinitely-long nanowires, which is divided into two parts. The first part is


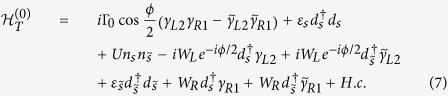


The relevant parameters here are defined as follows: 
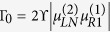
, 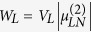, and 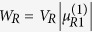 in which 
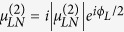
, 
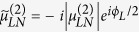
, 
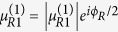
, and 
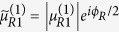
 (It is reasonable to suppose 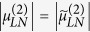 and 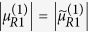). Besides, in the above formula 
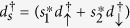
, 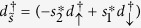, and 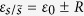 with 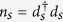 and 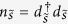.

For the second part, when the highest-order terms are neglected, it can be approximated as


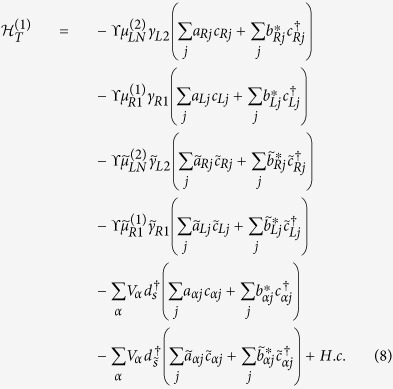


We would like to emphasize that since the *s*-wave pairing is present in the quantum wires, the electrons *c*_*α*_ and 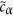 will form a Cooper pair and condense. This process leads to an effective coupling between Majorana zero modes localized at the same end and the finite coupling between the Kramers doublet in the QD. Therefore, up to the second-order perturbation in the tunneling process, we can express 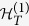 as


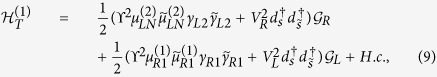


where 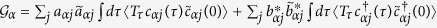 with 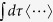 being a time-ordered integral. This process arises from the fact that the second-order perturbation can be treated as a Green function from the Lehnmann’s representation viewpoint or equivalently handled by the path-integral approach with defining one propagator. More detailed deduction can be referred in the previous works^31^,^32^,^35^. In the case of uniform superconducting pairings in the Majorana nanowires, 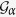 can be further deduced as 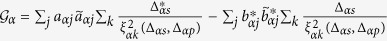 in which *ξ*_*αk*_ is the eigen-energy of the isolated superconductor. With the relations in Eq. (6), we can get the relationship that 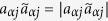 and 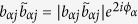. Accordingly, 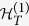 can be written as


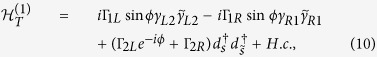


in which 
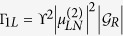
, 
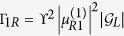
, and 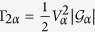. Up to now, we have gotten the low-energy effective Hamiltonian of such a structure. Noted, additionally that though there could be an additional coupling via the bulk superconductor to which both wires are proximity-coupled, such term is avoidable if wires are placed on two different superconductors^36^.

The phase difference between the two Majorana wires will drive finite Josephson current through them, which can be directly evaluated by the following formula^37^


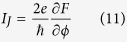


where 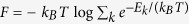 denotes the free energy with *E*_*k*_ and *T* being the junction’s eigen-energy and temperature. It is certain that solving the Josephson current is dependent on the diagonalization of 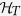.

In the following, we try to diagonalize the Hamiltonian. To start with, by defining 
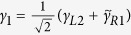
 and 
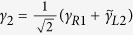
 with 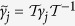, we reexpress *H*_*T*_ as


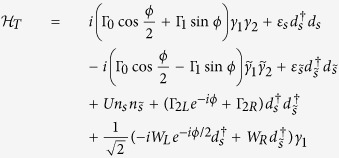



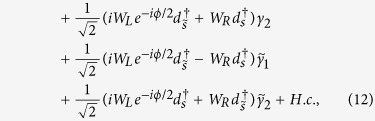


where Γ_1*α*_ is supposed to be Γ_1_. Next, 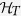 can be expressed in the normal fermion representation by supposing *γ*_1_ = (*f* + *f*^ †^), *γ*_2_ = *i*(*f*^ †^ − *f*  ) and 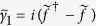, 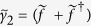 where *f*^ †^, 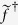 and *f*, 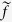 are the fermionic creation and annihilation operators. Accordingly, the matrix form of 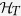 can be deduced on the basis of 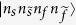 where *n*_*f*_ = *f*^ †^*f* and 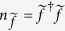. Note that in the system with Majorana bound states, only FP is the good quantum number, so we should build the Fock state according to FP. First, in the case of even FP, the Fock state can be written as 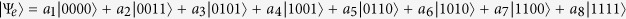. As a result, the matrix form of 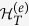 can be given by


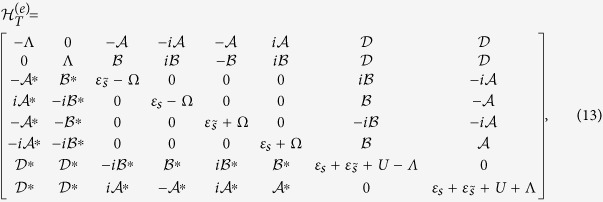


where 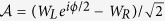, 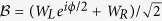, 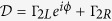, 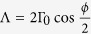, and Ω = 2Γ_1_sin*ϕ*. Next, for the case of odd FP, the Fock state can be written as 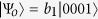

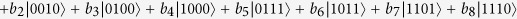
and the matrix of 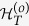 takes the form as


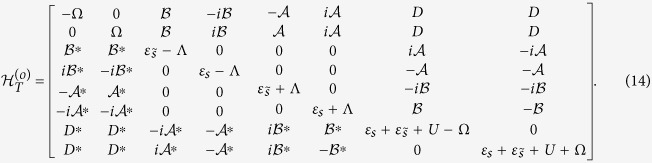


For the extreme case of strong magnetic-field limit, if 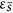 is in the finite-energy region, *ε*_*s*_ will be empty, and then only one level contributes to the Josephson effects, respectively. Accordingly, in such a case, the matrixes of 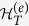 and 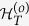 will be halved, i.e.,


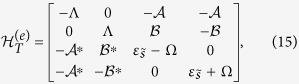


and


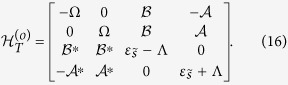


With the help of the above analysis, we know that the Josephson current should be evaluated by calculating the free energy according to FP, i.e., 
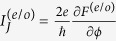
. In the zero-temperature limit, the Josephson current in this structure will get its simplified form as


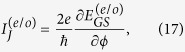


in which 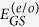 are the ground-state (GS) energies in the even- and odd-FP cases, respectively.

## Numerical Results and Discussions

Following the derivation in the above section, we begin to perform the numerical calculation to discuss the detailed properties of the Josephson current through such a system. For describing the Josephson effect governed by Majorana doublets, the parameter order should be much smaller than the superconducting gap Δ_*TS*_, hence we assume the parameter unit to be 0.1Δ_*TS*_ without loss of generality. In addition, for temperature, we will set it to be zero in the context.

Let us first review the Josephson effect in the case of *V*_*α*_ = 0. In such a case, 
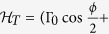
 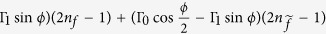, and 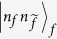 are the eigenstates of 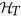. The two even-FP eigenstates are 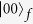 and 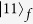, and their corresponding GS energies are 
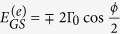
. Contrarily, the odd-FP eigenstates are 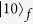 and 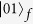 with the GS energies 
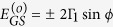
. Just as concluded in the previous works^32^, the fractional Josephson effect occurs in the situation of even FP, otherwise only the normal Josephon effect is observed.

**Figure 2:**
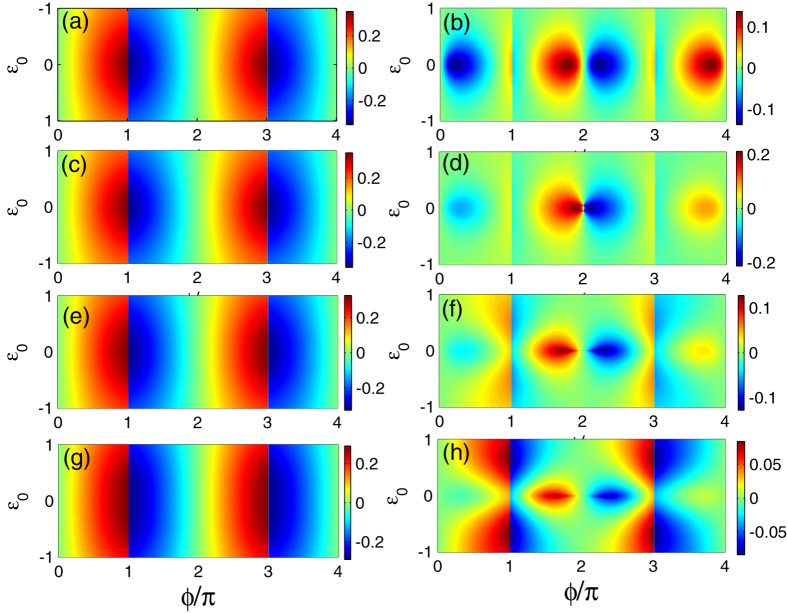
Josephson current spectra in the case where the Majorana doublets couple indirectly via a QD. The structural parameters are taken to be *W*_*α*_ = 0.25 and Γ_2*α*_ = 0.05. The left and right columns correspond to the even-FP and odd-FP results, respectively. (**a**,**b**) The case of the non-magnetic QD. (**c**,**d**) The case of finite magnetic field on the QD with *R* = 0.1. (**e**,**f**) *R* = 0.3. (**g**,**h**) *R* = 0.5.

**Figure 3:**
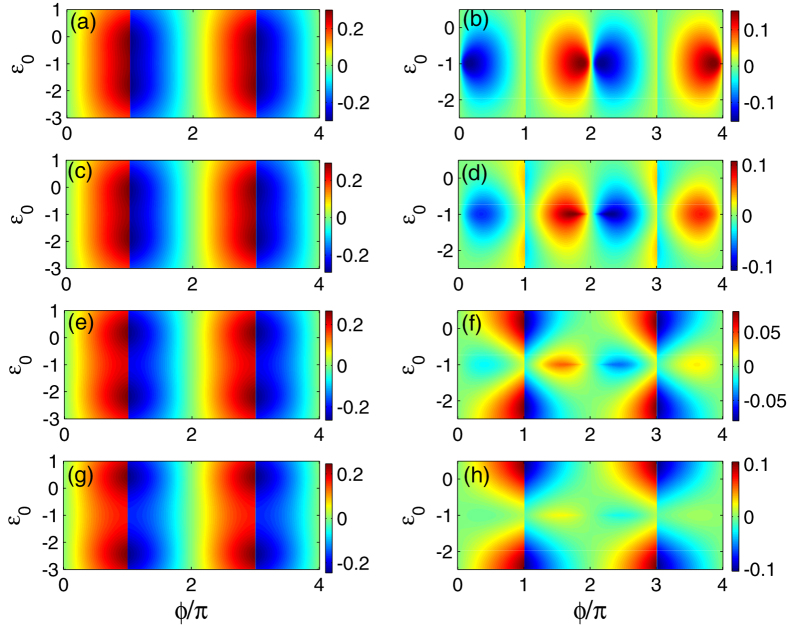
Josephson current in the case where the Majorana doublets couple indirectly via a QD of finite Coulomb interaction. The Coulomb strength is *U* = 2.0 and the others are the same as those in Fig. 2. The left and right columns correspond to the even-FP and odd-FP results, respectively. (**a**,**b**) The case of the non-magnetic QD. (**c**,**d**) The case of finite magnetic field on the QD with *R* = 0.1. (**e**,**f**) *R* = 0.3. (**g**,**h**) *R* = 0.5.

In Fig. 2 we suppose Γ_0_ = Γ_1_ = 0 and choose *W*_*α*_ = 0.25 and Γ_2*α*_ = 0.05 to investigate the Josephson effect in the case where the Majorana doublets couple indirectly to each other via a QD. The results are shown in Figs 2, 3: Fig. 2 corresponds to the noninteracting results, and Fig. 3 describes the influences of the intradot Coulomb interaction on the Josephson effects in different FPs when *U* = 2.0. First, in Fig. 2(a,b) we find that when a non-magnetic QD is taken into account, it induces the occurrence of normal Josephson effects, and the departure of *ε*_0_ from zero weakens the current amplitudes, irrelevant to the FP difference. In addition, FP is an important factor to affect the Josephson effects. To be concrete, the Josephson currents in different FPs flow in the opposite directions for the same *ϕ*, and the amplitude of 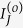 is about one half of that of 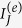. Moreover when |*ε*_0_| > 0.5, 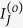 gets close to zero. The other result is that at the points of *ϕ* = (2*m* − 1)*π* (*m* ∈ *Integer*), in the even-FP case the discontinuous change of the Josephson current is more well-defined compared with the odd-FP case. Next, when finite magnetic field is applied on the QD, the even-FP current shows little change except that its amplitude becomes less dependent on the QD-level shift. However, in the odd-FP case, the current changes completely. It can be clearly found that with the strengthening of magnetic field, the original current oscillation is suppressed. Especially in the vicinity of *ϕ* = 4*mπ*, the current amplitude tends to disappear when *R* increases to 0.5. Thus, it is certain that in the case of odd FP, applying magnetic field on the QD can induce the occurrence of fractional Josephson current. In addition to this, the increase of *R* enhances the current oscillation around the points of *ϕ* = (2*m* − 1)*π* when *ε*_0_ departs from zero. Up to now, we can conclude that when the Majorana doublets are coupled by a magnetic QD, the fractional Josephson effect has an opportunity to take place, but its property is different from the case of only the direct coupling between Majorana doublets^32^.

Coulomb interaction is a key factor to influence the characteristics of QD. In Fig. 3 we consider the intradot Coulomb interaction and investigate the effect of the magnetic QD on the Josephson currents in the case of *U* = 2.0. In Fig. 3(a,b) we first find that in the even-FP case with a non-magnetic QD, the Coulomb interaction benefits the Josephson effect, since in the region of −2.5 < *ε*_0_ < 0.5 the current amplitude is relatively robust and weakly dependent on the shift of QD level. In contrast, for the odd-FP case, the intradot Coulomb interaction only moves the current maximum to the point of *ε*_0_ = −1.0, but it does not vary the current oscillation manner compared with the noninteracting case. Hence, the Coulomb interaction only adjusts the effect of QD level on the Josephson effects but does not modify the current oscillation manner with the change of *ϕ*. Next, Fig. 3(c,h) show that regardless of the FP difference, the current amplitudes are suppressed by the application of magnetic field on the QD. In the even-FP case, the current amplitude around the point of *ε*_0_ = −1.0 undergoes a relatively-apparent suppression. For the odd-FP case, except the suppression of the current amplitude, the fractional Josephson effect becomes weak but can still be observed.

According to the results above, when Majorana doublets couple indirectly via a magnetic QD, the fractional Josephson effect comes into being in the odd-FP case. In order to explain this phenomenon, we would like to compare the case of *ϒ* ≠ 0 and *V*_*α*_ = 0 with that of ϒ = 0 and *V*_*α*_ ≠ 0. In the former case, the Josephson effects are only determined by the FP of state 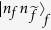. And then, when the system is located at states 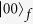 or 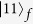, the fractional Josephson effect takes place. However, when Majorana doublets couple indirectly via one QD, the Fock space defined by 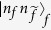 just becomes a subspace of the Fock space formed by 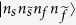, and then 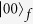 and 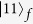 appear simultaneously in the expressions of 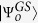. This means that when their contributions are different, fractional Josephson effect will have an opportunity to take place. It can be found that in the odd-FP case, this condition can be satisfied in the case of nonzero magnetic field. The reason is as follows. Firstly, magnetic field on the QD can cause the occupation of opposite-spin electrons to be different, e.g., 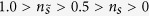 in the case of *ε*_0_ = 0. This will effectively enhance the amplitudes of *b*_3_ and *b*_5_ and the contribution of states 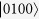 and 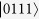. Secondly, Eq. (14) shows that these two states couple to any other state in an asymmetric manner. As a result, if finite *R* is considered, 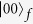 and 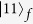 make different contributions to the Josephson effect, leading to the fractional Josephson effect. Surely one can find that the asymmetric coupling manner between the basis states weakens the quantum coherence and suppresses the current amplitude to some degree. Next in the presence of Coulomb interaction, the QD is half-occupied at the point of *ε*_0_ = −*U*2, so the fractional Josephson effect occurs in the case of *ε*_0_ = −1.0 when *U* = 2.0. With the similar analysis method, one can understand the Jopsephson effect in the even-FP case, and its noninteracting picture can only be doubled when the Coulomb interaction is taken into account.

**Figure 4:**
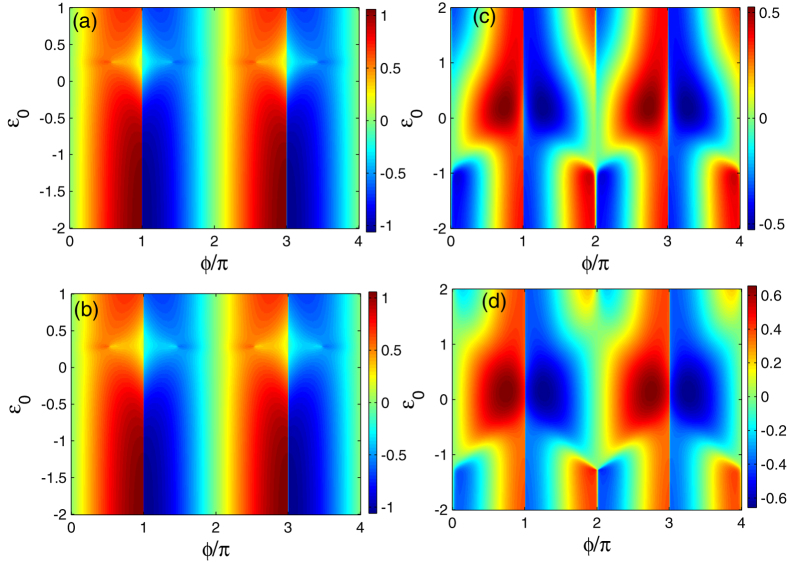
Josephson current spectra in the case where the direct and indirect couplings between the Majorana doublets co-exist. The relevant parameters are Γ_0_ = 0.5, Γ_1_ = 0.1, *W*_*α*_ = 0.25, and Γ_2*α*_ = 0.05. (**a**,**b**) The even-FP case with non-magnetic and magnetic QDs with *R* = 0.3. (**c**,**d**) The odd-FP results with *R* = 0 and *R* = 0.3.

**Figure 5:**
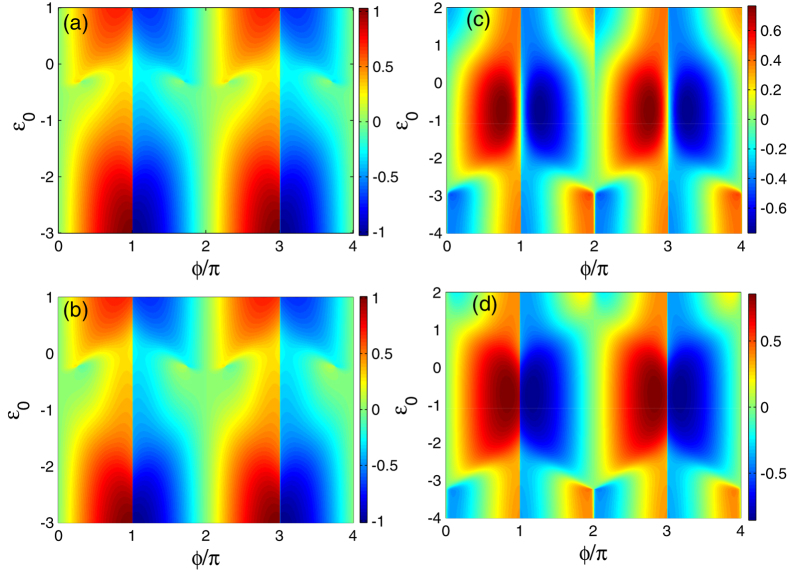
Josephson current spectra in the case of simultaneous direct and indirect couplings between the Majorana doublets. The Coulomb strength is fixed at *U* = 2.0, and the other parameters are identical with those in Fig. 4. (**a**,**b**) The even-FP case with non-magnetic and magnetic QDs with *R* = 0.3. (**c,d**) The odd-FP results with *R* = 0 and *R* = 0.3.

We next proceed to pay attention to the Josephson effect in the case where the direct and indirect couplings between the Majorana doublets coexist. The results are shown in Figs 4 and 5 where Γ_0_ is taken to be 0.5 with Γ_1_ = 0.1. The noninteracting results are presented in Figs 4 and 5 describes the case of *U* = 2.0. In Fig. 4, we find that for any *ε*_0_, the opposite-FP Josephson currents show dissimilar oscillations with the change of superconducting phase difference. In the even-FP case with *R* = 0, when *ε*_0_ gets approximately close to 0.25, the amplitude of Josephson current decreases, otherwise, the Josephson effect will be enhanced and then holds. In such a process, the current oscillation manner does not change [See Fig. 4(a)]. Next, in Fig. 4(b) where *R* = 0.3, we can see that the only effect of the magnetic field is to suppress the minimum of the Josephson current. Such a result is similar to the case of Γ_0_ = 0. On the other hand, for the odd-FP case, Fig. 4(c) shows that in the region of *ε*_0_ < −1.0, the Josephson current tends to oscillate more seriously. And when the QD level increases to be *ε*_0_ = −1.0, the current period seems to experience the discontinuous (*π* → 2*π*)-like transition followed by the current disappearance near the points of *ϕ* = 2*mπ*. Next, in the region of −1.0 < *ε*_0_ < 1.0, the Josephson current oscillates weakly with the change of *ϕ*, and its maximum appears in the vicinity of *ε*_0_ = 0. When *ε*_0_ further increases from 1.0, the Josephson current recovers its form of *ε*_0_ < −1.0 gradually. Figure 4(d) presents the effect of the magnetic field on the QD in odd-FP case. It seems that in such a case, the magnetic field cannot induce the fractional Josephson effect, but it tends to enhance the current amplitude in the region of −1.0 < *ε*_0_ < 1.0, which is exactly opposite to the case of Γ_0_ = 0.

Following the above result, we present the influence of the magnetic field on the case of finite Coulomb interaction. The results are displayed in Fig. 5, where the Coulomb strength is also taken to be *U* = 2.0. Firstly, Fig. 5(a) shows the even-FP result with the non-magnetic QD. We can find that in such a case, the current minimum shifts to the position of *ε*_0_ ≈ −0.25. Besides, the Coulomb interaction efficiently weakens the Josephson effect, since increasing *ε*_0_ from −2.0 begins to eliminate the current amplitude gradually. Next when magnetic field is applied on the QD with *R* = 0.3, it further suppresses the minimum of the Josephson current, similar to the noninteracting case [See Fig. 5(b)]. The odd-FP results are shown in Fig. 5(c,d) with the magnetic field strength being zero and 0.3, respectively. In Fig. 5(c), we see that differently from the noninteracting result, the 2*π*-period oscillation of the current occurs from *ε*_0_ = −3.0. In the region of −3.0 < *ε*_0_ < 1.0, the Josephson current varies in period 2*π* with its maximum in the vicinity of *ε*_0_ = −1.0. Besides, it can be noted that Coulomb interaction enhances the amplitude of the Josephson current, in comparison with the noninteracting results. For the effect of magnetic field in the odd-FP case, as shown in Fig. 5(d), it is analogous to that in the noninteracting case. Namely, it tends to enhance the current amplitude in the region of −3.0 < *ε*_0_ < 1.0 but does not induce the appearance of fractional Josephson effect.

**Figure 6:**
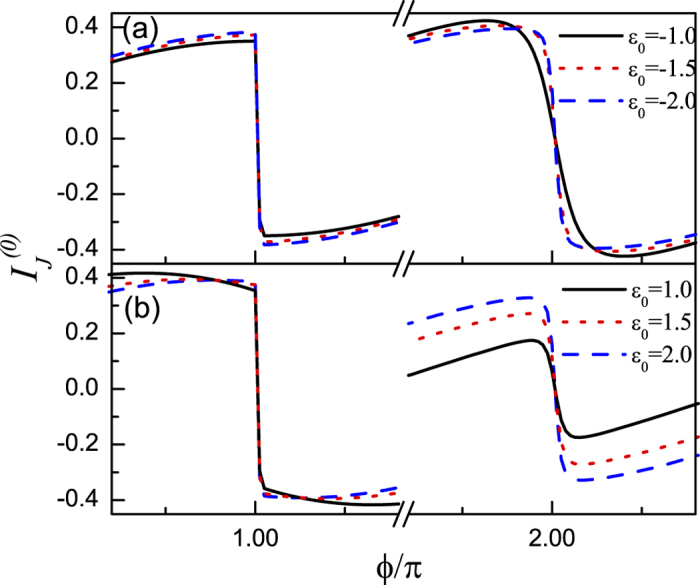
Odd-FP Josephson current in the presence of direct and indirect couplings between Majorana doublets. In (**a**) *ε*_0_ = −1.0, −1.5, −2.0, and *ε*_0_ = 1.0, 1.5, 2.0 in (**b**). The other parameters are the same as those Fig. 4.

The results in Figs 4 and 5 can be explained following the discussion about Figs 2 and 3. In the case of nonzero *ϒ*, the underlying physics that governs the Josephson effects certainly becomes complicated. The reason arises from two aspects. Firstly, the fermion occupation in the QD re-regulates the FP of 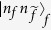 for conserving the FP of the whole system. Secondly, the Fano interference can be induced due to the direct and indirect couplings between the Majorana doublets. We notice that in the even-FP case without magnetic field, when the QD level is away from the energy zero point, both *n*_*s*_ and 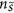 will be close to 1 or 0 simultaneously. This causes the even-FP states of 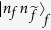, i.e., 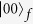 and 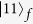, to co-contribute dominantly to the Josephson effect. It is known that at these two states, the 4*π*-periodic currents are direction-opposite, so the normal Josephson effect appears in Fig. 4(a) where the current amplitude is proportional to Γ_0_. Alternatively, in the odd-FP case with |*ε*_0_| > 1.0, states 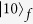 and 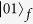 will make leading contribution to the Josephson effect. Thus, the *π*-period-like current arises with its amplitude related to Γ_1_. However, after observing the result in Fig. 6(a,b), one can find that the period of the odd-FP current is still 2*π* even in the case of *ε*_0_ ≤ −1.0, because the current profiles near the point *ϕ* = *π* and *ϕ* = 2*π* are different. On the other hand, when the QD level gets close to the energy zero point, it will become half-occupied. In such a situation, 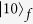 and 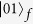 contribute to the even-FP Josephson current, whereas 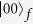 and |11〉_*f*_ devote themselves to the odd-FP current. However, due to Γ_1_ ≪ Γ_0_, the suppression of 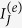 only appears in a narrow region near the point of *ε*_0_ = 0, while the 2*π*-periodic oscillation of 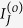 distributes in a wide region accompanied by its enhanced amplitude [See Fig. 4(a,c)]. In what follows, in the presence of intradot Coulomb interaction, *ε*_*s*_ splits into two, i.e., *ε*_*s*_ and *ε*_*s*_ + *U*. As a consequence, in the energy region of −*U* < *ε*_*s*_ < 0, the fermion in the QD is changeable between 0 and 1, which magnifies the transformation of the Josephson effect caused by the shift of QD level. Since the magnetic field and Coulomb interaction play similar roles in affecting the fermion occupation in the QD, their influences on the Josephson current are also analogous to each other. In addition, it is worth noticing that the Fano interference induces the asymmetric spectra of the Josepshon currents vs *ε*_0_.

**Figure 7:**
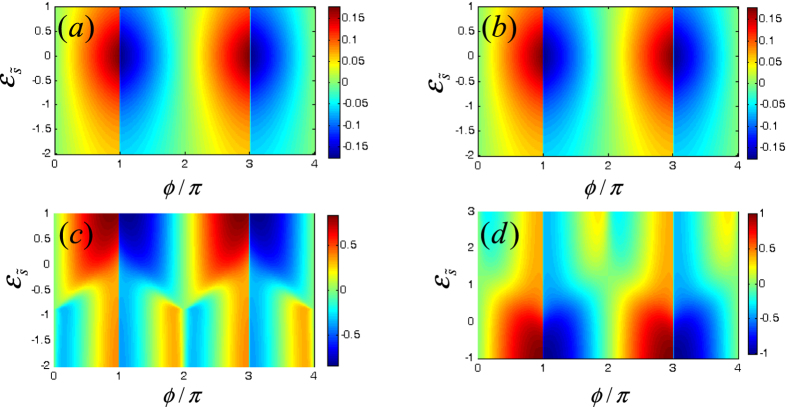
Josephson currents in the limit of strong magnetic field on the QD. The left and right columns describe the even and odd FP results. (**a**,**b**) The cases of Γ_0_ = 0. (**c**,**d**) Results of Γ_0_ = 0.5.

At last, we focus on the extreme case of strong magnetic field where only one level (i.e., 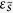) contributes to the Josephson effects. In such a case, the matrix dimension of 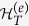 and 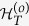 will be halved, as discussed in the above section. The corresponding numerical results are shown in Fig. 7. In Fig. 7(a,b) we can see that in the case of Γ_0_ = 0, the Josephson currents in different FPs are the same as each other, with their period 2*π*. On the other hand, when the direct coupling between the Majorana doublets is considered, the Josephson currents become dependent on FP. As shown in Fig. 7(c), in the even-FP case, increasing 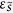 can change the current oscillation with the clear transition region near 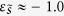. Instead, in the odd-FP case, similar result occurs when 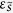 decreases. These results can certainly be clarified by discussing the influence of the fermion number in the QD on the FP of states 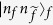. Additionally, in Fig. 7(c,d) we find that the Fano interference leads to the dissimilar transition behaviors of the Josephson currents for different FPs.

## Summary

In summary, we have investigated the Josephson effect contributed by the Majorana-doublets via considering the different inter-doublet coupling manners. It has been found that an embedded QD in this junction plays a nontrivial role in modifying the Josephson currents, since the tunable fermion occupation in the QD re-regulates the FP of the Majorana doublets for conserving the FP in whole system. As a result, the 4*π*-period, 2*π*-period, and *π*-period-like Josephson currents have opportunities to come into being, respectively, following the change of structural parameters. To be concrete, when the Majorana doublets couple indirectly via a non-magnetic QD, the normal Josephson effects occur, and the FP change just leads to the reversal of current direction and the variation of current amplitude. With the application of magnetic field on the QD, the fractional Josephson effect comes into being in the situation of odd FP. On the other hand, when the direct and indirect couplings between the Majorana doublets coexist, no fractional Josephson effect takes place, regardless of finite magnetic field on the QD. Instead, there almost emerges the *π*-period-like current with the shift of QD level in the odd-FP situation. In addition to the above results, it showed that compared with the magnetic field and inter-doublet coupling manner, the effect of Coulomb interaction on the Josephson current is relatively weak. All the results have been clarified by analyzing the contributions of respective basis in the Fock space. We believe that this work will be helpful for describing the QD-assisted Josephson effects between Majorana doublets.

We also would like to emphasize the experimental feasibility of our considered Josephson junction. Firstly, according to the experimental and theoretical progresses, the one-dimensional DIII-class TS can be fabricated in different ways^23^,^38^,^39^,^40^. This is very important for the achievement of such a junction. Secondly, the QD fabrication is very sophisticated, and the QD-related parameters can be well controlled by adjusting the gate voltage and QD size^41^. Therefore, we consider that the main results in this work can be experimentally realized with high feasibility.

## Additional Information

**How to cite this article**: Gong, W.-J. *et al*. Influence of an embedded quantum dot on the Josephson effect in the topological superconducting junction with Majorana doublets. *Sci. Rep*. **6**, 23033; doi: 10.1038/srep23033 (2016).
